# Case Report: Exercise-associated changes of leukocyte gene expression in statin-associated myopathy

**DOI:** 10.3389/fphar.2025.1695543

**Published:** 2025-12-04

**Authors:** Galyna Bondar, Abhinandan Das Mahapatra, Irina Silacheva, Tra-Mi Bao, Thomas Vu, Stephanie Su, Adrian Hairapetian, Ananya Katappagari, Liana Galan, Joshua Chandran, Ruben Adamov, Alan Yang, Ananya Bukkapatnam, Pejman Mansouri, Mahi Mirchandani, Nathan Dang, Lorenzo Mancusi, Isabel Lai, Anca Rahman, Tristan Grogan, Jeffrey Hsu, Monica Cappelletti, PeiPei Ping, David Elashoff, Elaine F. Reed, Mario C. Deng

**Affiliations:** 1 David Geffen School of Medicine, University of California Los Angeles Medical Center, Los Angeles, CA, United States; 2 Associate Bioinformatics Engineer III, Organization: Strand NGS, Strand Life Sciences Pvt. Ltd., Bengaluru, India

**Keywords:** statin-associated myopathy, immunological fitness, exercise-associated changes, statin-associated adverse effects, statin-myopathy, peripheral blood mononuclear cell transcriptome profiling

## Abstract

**Background:**

Statin-associated muscle symptoms (SAMS) are a significant clinical issue, and their exact cause is not well understood. Immunological mechanisms have been suggested but have not been confirmed. This study is a rare, longitudinal case-based analysis that uses transcriptomics to explore immune-related gene expression changes in peripheral blood mononuclear cells (PBMCs) in response to exercise before, during, and after the onset and resolution of SAMS.

**Methods:**

A healthy volunteer (HV1) enrolled in an exercise immuno-fitness study underwent cardiopulmonary exercise testing (CPX) with blood collected at three timepoints: pre-exercise (TP1), peak exercise (TP2), and 1 hour post-exercise (TP3). After baseline testing (Visit 1), the participant began statin therapy on their own, developed SAMS, and had repeat CPX testing during the symptomatic phase (Visit 2) and partial recovery phase (Visit 3). RNA was extracted from PBMCs and analyzed using next-generation RNA sequencing. The data were evaluated using differential gene expression analysis and Weighted Gene Co-expression Network Analysis (WGCNA). Pathway and gene ontology enrichment were used to identify immunologic signatures associated with SAMS.

**Results:**

The PBMC gene expression profiles showed distinct changes during SAMS compared to the baseline and recovery phases. WGCNA identified 39 co-expression modules. Several modules had high expression at peak exercise in the healthy state (V1), which was attenuated in SAMS (V2) and partially restored in recovery (V3). Gene ontology and Reactome analyses of key modules identified 16 genes that were differentially expressed at peak exercise and may be involved in specific immune pathways in SAMS pathogenesis.

**Conclusion:**

This case study suggests that profiling the exercise-induced immune transcriptome can reveal dynamic immunological changes related to statin-induced myopathy. These findings support the hypothesis of an immune-mediated component in SAMS and provide a basis for future studies to validate transcriptomic biomarkers for the early detection and management of SAMS.

## Introduction

The drug class of 3-hydroxy-3-methyl-glutaryl coenzyme A (HMG-CoA) reductase inhibitors, also called statins, represent the first line therapy in the prevention and treatment of cardiovascular diseases (CVD) which are considered the primary causes of mortality in the world (Camarino et al., 2021). Statin-associated muscle symptoms (SAMS), ranging from mild myalgia to life-threatening rhabdomyolysis, cover a wide range of phenotypes (Stroes et al., 2015). Their precise classification continues to be debated. Different hypotheses to understand the causes of SAMS including depletion of cholesterol in muscle cell membranes, reduction of intermediates of the cholesterol biosynthetic pathway, reduction of seleno-protein synthesis, inhibition of AKT/mTOR signaling pathways, modification of ion-channel conductance, genetic disposition, modulation of mitochondrial function and initiation of an aberrant immuno-myopathy are being entertained but a knowledge gap about definitive causation of SAMS continues to persist (Camarino et al., 2021). We describe a well-phenotyped case that sheds light on the immunological hypothesis of SAMS-causation.

## Methods and design

This study stems from a larger “Exercise-associated Changes of Immune-Fitness in Heart Failure” study that included 16 heart failure subjects and four healthy volunteers (Bondar et al., 2024). It is based on our prior molecular prediction test development experience with heart transplant rejection (Deng et al., 2006; Deng 2021), heart failure (HF) survival (Bondar et al., 2017; Deng 2018; Bondar et al., 2021) and Long Covid Outcome (Deng 2020).

### Case-description

**FIGURE 1:**
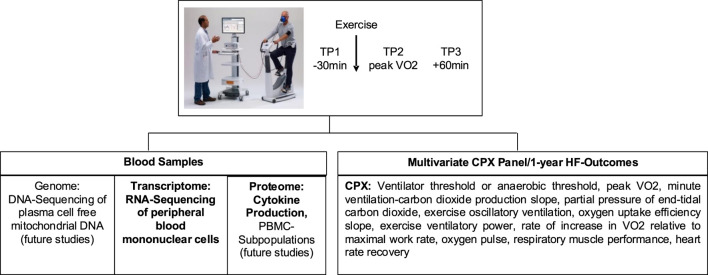
Flowchart describing study protocol (reprinted from Bondar et al., 2024, licensed under CC BY 4.0)

Healthy volunteer 1 (HV1), who was also the Principal Investigator, underwent the study’s protocol (Figure 1) which included Cardiopulmonary Exercise Testing (CPX) with blood sampling at three time points: baseline (TP1), peak exercise (TP2), and 1 h after exercise (TP3) on 2 March 2018 (Visit 1 = V1).

On 4 October 2019, HV1 independently started high dose atorvastatin (40 mg) for preventative cardiology, following 2018 AHA/ACC clinical guidelines predicting a 10-year arteriosclerosis/cardiovascular disease (ASCVD) of 7.3% with optimal risk factor management. Over the next few weeks, HV1 who was an avid jogger in excellent physical fitness condition with a past medical history of a singular event of cryptogenic transitory ischemic attack in 2007, longstanding and well-controlled arterial hypertension and history of lone atrial fibrillation event in 1989 as well as duodenal ulcer in 2005 developed SAMS. These symptoms consisted of bilateral lower extremity leg pain and weakness that affected gait, for example, requiring frequent breaks during inpatient rounds in the hospital and impairing foot muscle extension (tip-toe walking). From the subject’s perspective the frequent “need to crouch down” was most disabling, along with secondary effect of worsening of a pre-existing lumbar spinal stenosis symptom complex including multilevel lumbar degenerative changes with spinal stenosis at L3-L4, L4-L5 and L5-S1. These symptoms led to the discontinuation of atorvastatin on 10 January 2020. The subject opted not to initiate statin replacement therapy.

The subject is a single child. His father was Chinese, suffered from hypertension and died at age 61 years from stroke. His mother was German, suffered from duodenal ulcers and was at the time of the initial SAMS-presentation in good health at 95 years of age. The subject has two healthy children, is married and works as clinician-scientist in a university hospital.

In addition, autoimmune etiology workup such as anti-HMG-CoA reductase myopathy testing for LD, aldolase and metabolic workup including TSH, vitamin D, electrolytes was completed and HMG-CoA reductase antibodies, antinuclear antibodies and myomarker 3+ panel were negative. Myopathy-associated electromyographic and nerve-conduction abnormalities were documented.

Therapeutically, physical therapy and anti-inflammatory prednisone therapy with a maximum initial dose of 10 mg/day prednisone and a 6 weeks tapering protocol were initiated. Following therapy with physical therapy and prednisone taper, SAMS symptoms slowly and partially improved. While the CK-levels trended down over the course of V3, some CK-elevation persisted, concordant with persistence of residual symptoms. In June 2023 and increasing recovery from SAMS-symptoms, HV1 was able to resume tennis training lessons. During the entire period, HV1 continued his professional work to the full extent.

Due to the unique opportunity to gain insights into the immunological pathogenesis of SAMS, the research team repeated the exercise test with the same protocol on 31 January 2020 (Visit 2 = V2) and again on 18 August 2022 (Visit 3 = V3).

### Blood sampling and processing

Samples were collected at 3 time points (TP): within 30 min before exercise (TP1), within 60 s of peak exercise (TP2), and within 1 h post-exercise (TP3) into 1 CPT (Becton Dickinson, Franklin Lakes, NJ, United States) tube for RNA-seq analyses. Eight mLl of blood was drawn into a CPT tube.

Peripheral Blood Mononuclear cells (PBMC) from each sample were purified within 2 h of phlebotomy. The collected blood was mixed and centrifuged at room temperature (22 °C) for 20 min at 2000× g RCF. Two mLl of plasma was separated without disturbing the cell layer into an Eppendorf tube (Sigma-Aldrich, St. Louis, MO, United States) and stored at −80 °C for future experiments. The cell layer was collected, transferred to 15 mL conical tubes, and re-suspended in cold Phosphate Buffer Saline (PBS) (Sigma-Aldrich, St. Louis, MO, United States) and centrifuged for 20 min at 300× g RCF at 4 °C. The supernatant was aspirated and discharged. The cell pellet was re-suspended in cold PBS, transferred into an Eppendorf tube, and centrifuged for 20 min at 300× g RCF at 4 °C. The supernatant was discharged. The pellet was re-suspended in 0.5 mL RNA Protect Cell Reagent (Qiagen, Valencia, CA, United States) and frozen at −80 °C. All samples were processed using next-generation RNA sequencing transcriptome analysis at the UCLA Technology Center for Genomics and Bioinformatics. Briefly, the RNA was isolated from the PBMC using RNeasy Mini Kit (Qiagen, Valencia, CA, United States). The quality of the total RNA was assessed using NanoDrop^®^ ND-1000 spectrophotometer (NanoDrop Technologies, Wilmington, DE, United States) and Agilent 2,100 Bioanalyzer (Agilent Technologies, Palo Alto, CA, United States) concentration above 50 ng/μL, purity—260/280–2.0, integrity—RIN >9.0 and average >9.5. Then, mRNA library was prepared with Illumina TruSeq RNA kit according to the manufacturer’s instructions (Illumina, San Diego, CA, United States). Library construction consists of random fragmentation of the polyA mRNA, followed by cDNA production using random polymers. The cDNA libraries were quantified using Qubit and size distribution was checked on Bioanalyzer 2,100 (Agilent Technologies, Palo Alto, CA, United States). The library was sequenced on HiSeq 2,500. Clusters were generated to yield approximately 725 K–825 K clusters/mm2. Cluster density and quality were determined during the run after the first base addition parameters were assessed. We performed single-end sequencing runs to align the cDNA sequences to the reference genome. Generated FASTQ files were transferred to the Advanced HF Research Data Center where Avadis NGS 1.5 (Agilent, Palo Alto, CA, United States and Strand Scientific, San Francisco, CA, United States) was used to align the raw RNA-Seq FASTQ reads to the reference genome. After RNA extraction, quantification and quality assessment, total mRNA was amplified and sequenced on the whole-genome Illumina HiSeq 2,500. Data were then subjected to DeSeq normalization using NGS Strand/Avadis (v2.1 10 October 2014).

### Transcriptome analysis

The sequenced data from each visit and time points were aligned with human hg38 genome followed by RNA-Seq analysis using StrandNGS v4.0 software. Further, filter by expression (20th-100th percentile) were performed to retain by genes that have significant normalized signal values in all samples.

We aimed to construct mixed effects regression models for each candidate feature with terms for Visit, TP, and the Visit-TP interaction. This allowed us to test differential trends in expression signals across the exercise Visits and TPs. We used the Benjamini–Hochberg procedure to control the false discovery rate (FDR) at alpha 0.05. If significant interaction effects were found after FDR adjustment, post hoc comparisons at specific time points or between time points and Visits were estimated using model contrasts and summarized with 95% confidence intervals estimated from the model.

To better understand co-expression of genes and their biological role in patients with HF, we performed WGCNA and pathway analyses (Langfelder and Horvath, 2008). WGCNA provides a non-supervised systematic method to identify co-expressed genes and pathways. This approach offers a powerful way to reduce dimensionality while enhancing biological interpretability. The co-expressed modules and their respective eigen-genes will be interpreted in their relationship to clinical and biological features.

The biological relevance of the modules was deduced using gene ontology [Froehlich et al., 2007; Froehlich 2015) (GOSim) and pathway analysis (Strand NGS (Avadis)).

## Results

### Result 1: clinical data

During visit 1 (V1), HV1 was in full possession of health, reflected in a CPX-performance of 162% predicted VO2. In contrast, during V2 performed during the acute phase of SAMS, the impact of SAMS-related reduced muscle strength was reflected in a decrease of predicted VO2 to 116%. The subsequent CPX 2 years later (V3) showed partial recovery and to a 134% of predicted performance. The timeline of HV1 visits 1,2 and 3 and CPX-related clinical parameters are summarized in Table 1.

**TABLE 1:** Clinical parameters at each timepoint.

Clinical parameters	Visit 1 (V1)	Visit 2 (V2)	Visit 3 (V3)
Timeline	2 March 2018	31 January 2020	18 August 2022
Clinical parameter			
SAMS-status	—	+++	+
CK (units/ml)	n.a	507–1,443	1,184
Workload (Watts)	274	183	182
Peak VO2 (mL/kg/min)	41.3	28.8	31.4
Predicted (%)	162	116	134

### Result 2: gene expression profiles

**FIGURE 2:**
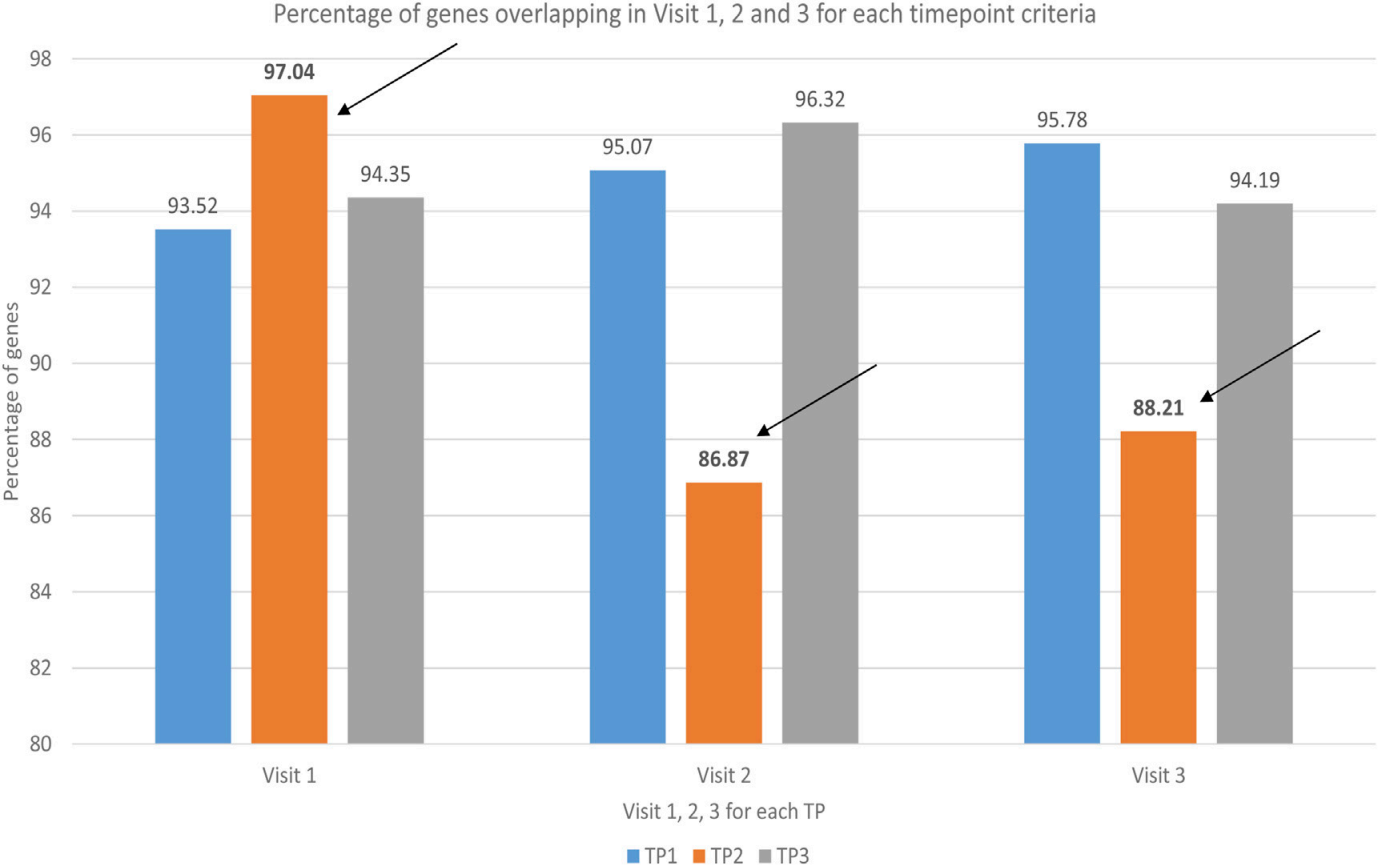
Bar diagram representing the comparison of % of overlapped genes in Visit 1, 2 and 3 for each timepoint. Under resting condition (timepoint 1-blue) and under recovery condition (timepoint 3-grey), more than 90% of PBMC genes overlapped in visit 1, visit 2 and visit 3. In contrast, at peak exercise (timepoint 2-orange, see arrows) less than 90% of PBMC genes overlapped in visit 2 and visit 3 (details see text).

Second, we analyzed PBMC-genes that fulfilled analysis inclusion criteria (20%–100% restriction, FDR correction p-value 0.05 and 2.0 – fold change across timepoints criteria) and overlapped for TP 1,2 and 3 in V1-3 (Figure 2). It was interesting that the first indication of SAMS-associated PBMC-biology changes were reflected in the reduced percent overlap percent overlap of expressed PBMC-genes, comparing V1 to V2 and V3.

### Result 3: WGCNA analysis

**FIGURE 3:**
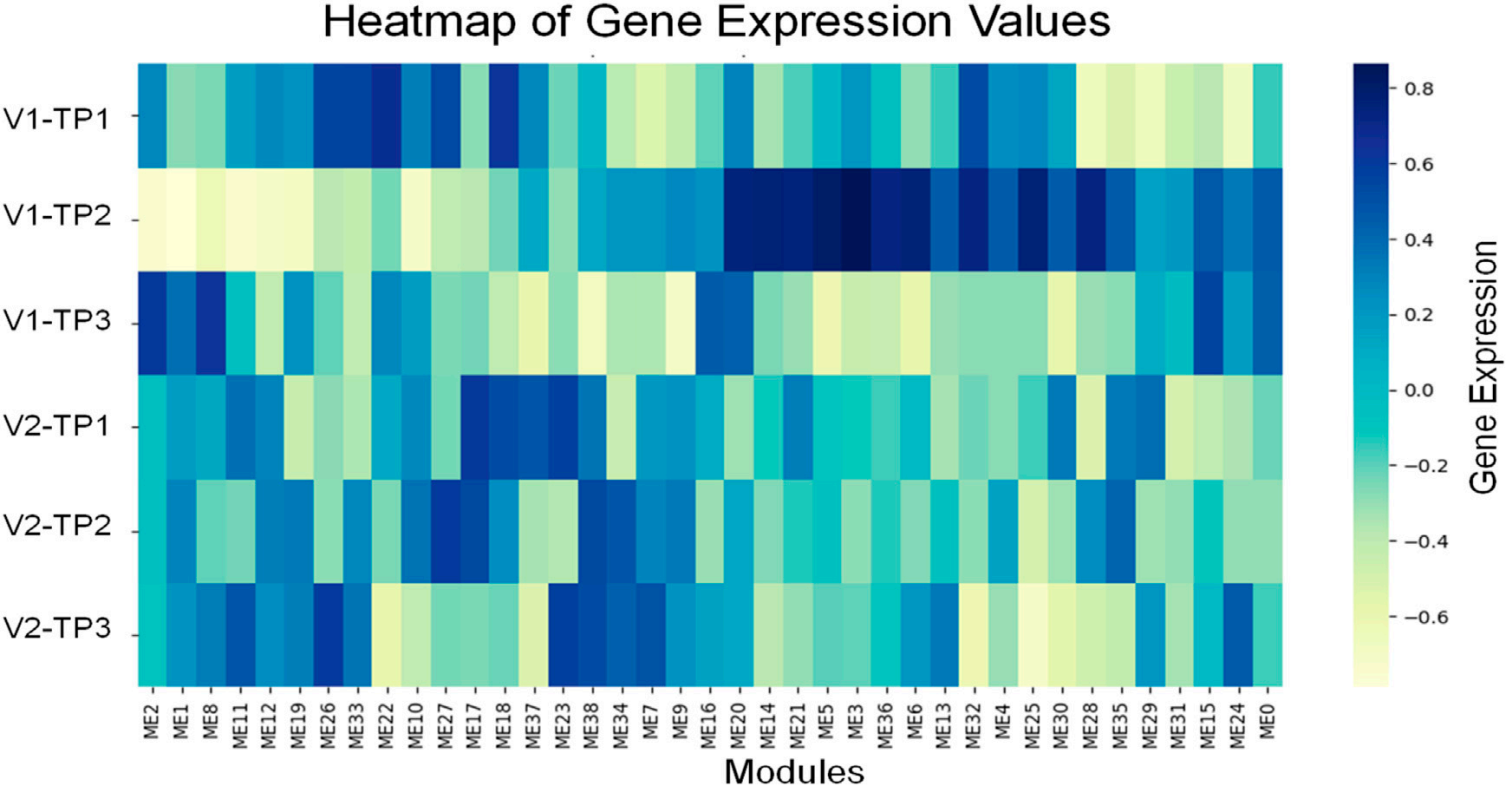
We identified 39 modules by WGCNA-analysis, represented by their module eigengenes (ME) (details see text).

Third, in order to reduce the complexity of PBMC-GEP in a biologically meaningful way, we performed WGCNA-analyses (Langfelder and Horvath, 2008). We identified 39 modules, represented by their module eigengenes (ME) (Figure 3).

### Result 4: module analysis

**FIGURE 4:**
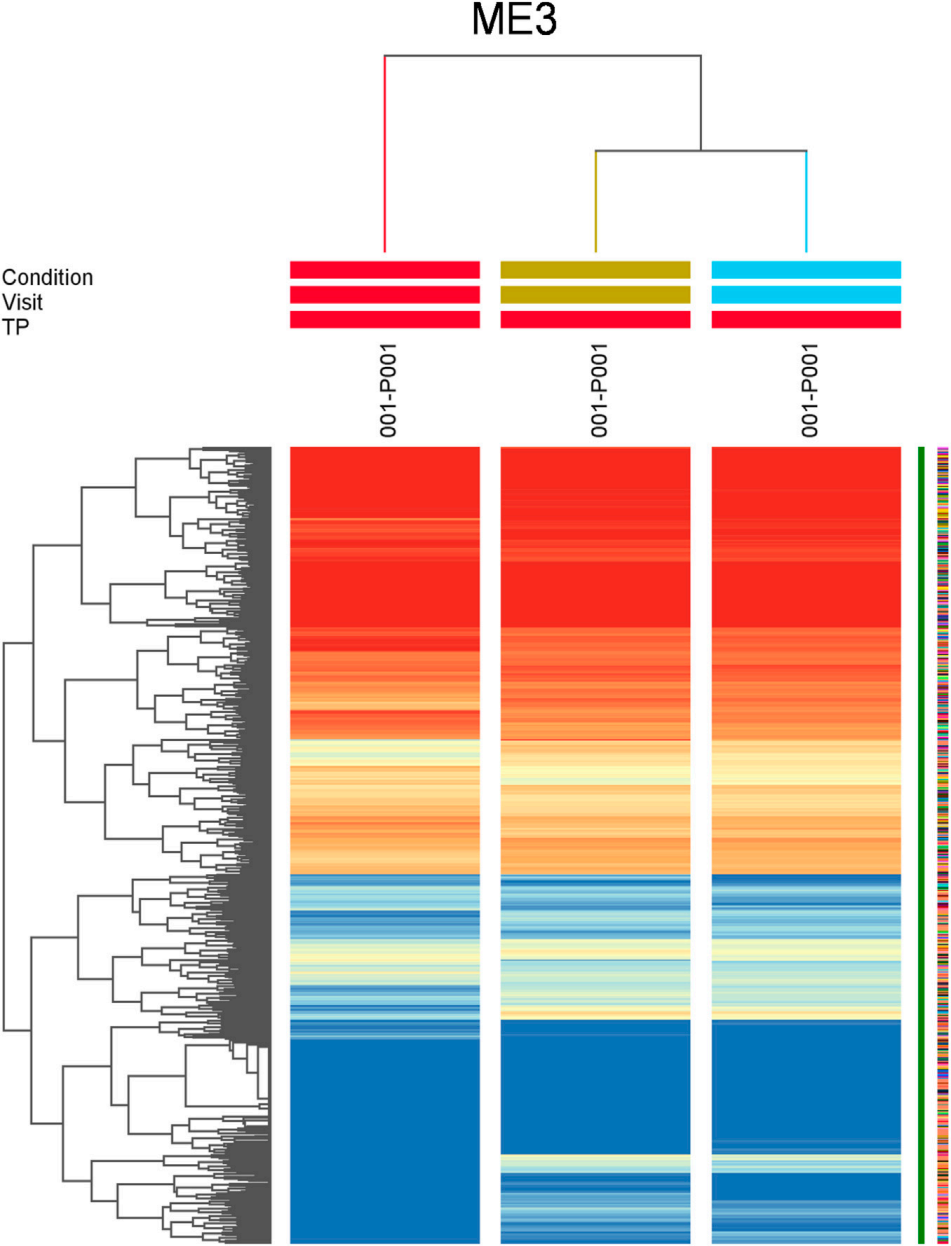
We analyzed modules by biological similarity using a clustering algorithm. Model eigengene 3 (ME3) at visit 1 (Health) separates from visit 2 (SAMS) and visit 3 (partial recovery). Similar results were obtained for ME5, 6, 14, 20, 21, 36 (details see text).

Fourth, we analyzed modules that showed the highest gene expression at TP2 in V1 (3, 5, 6, 14, 20, 21, 36) (full health) and grouped the ME by biological similarity, using clustering algorithm. Healthy state samples (V1) separated consistently from SAMS-state (V2) samples and partial recovery (V3) samples (Figure 4).

### Result 5: gene ontology and reactome analyses

**FIGURE 5:**
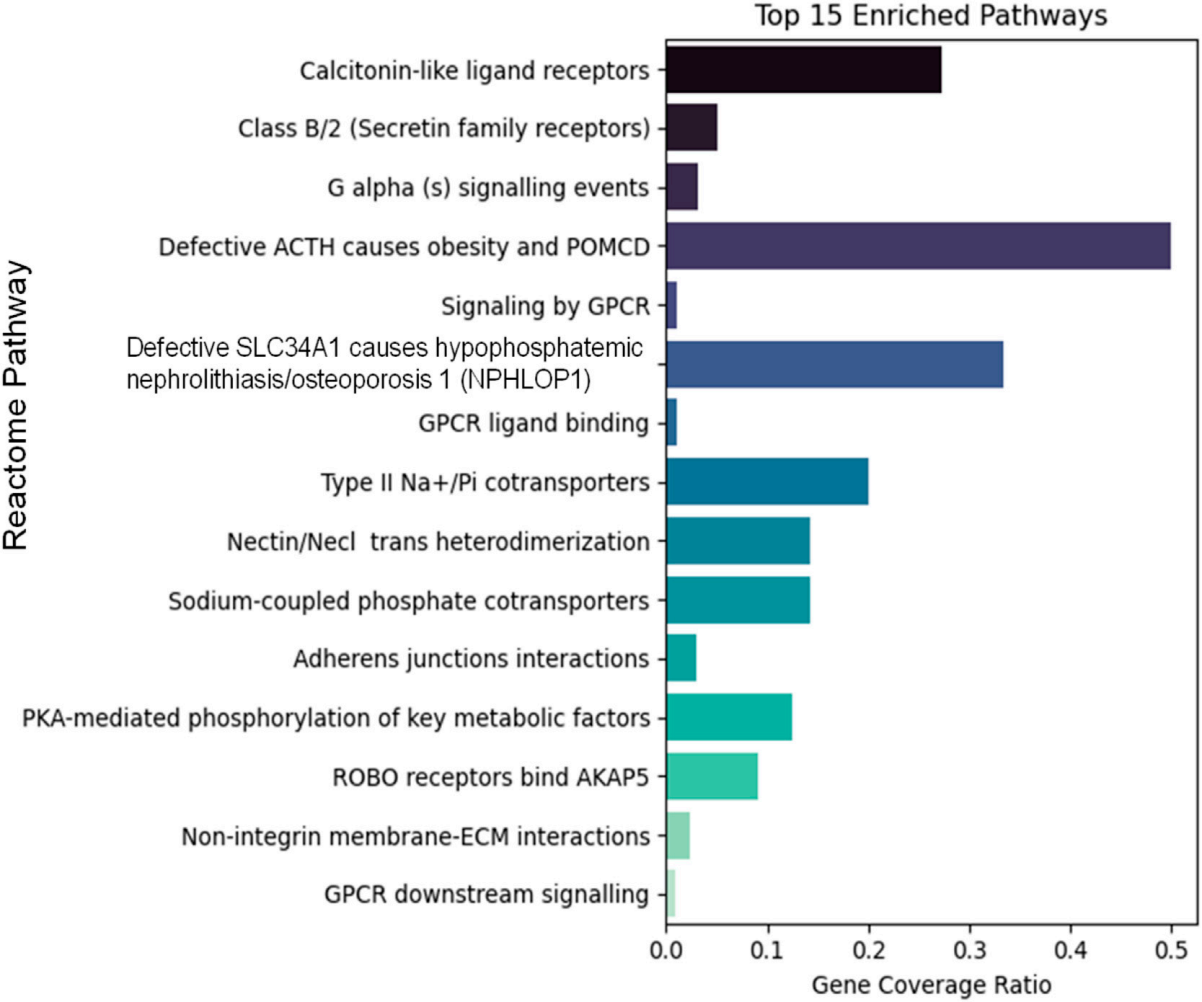
Reactome analysis of genes in module 3 that are differentially expressed at peak exercise in full health (visit 1, TP2) compared with SAMS (visit 2, TP2) (details see text).

Fifth, in order to investigate the potential role that the PBMC-genes in the high expressing modules exert in SAMS, we analyzed Gene Ontology- and Reactome patterns. For proof-of-concept demonstration, module three was analyzed with respect to genes that were differentially expressed at peak exercise in full health (visit 1, TP2) compared with SAMS (visit 2, TP2) and could therefore potentially attributed to statin-associated mediation of SAMS (Figure 5) Hier gehts weiter.

### Result 6: pathway analysis

**FIGURE 6:**
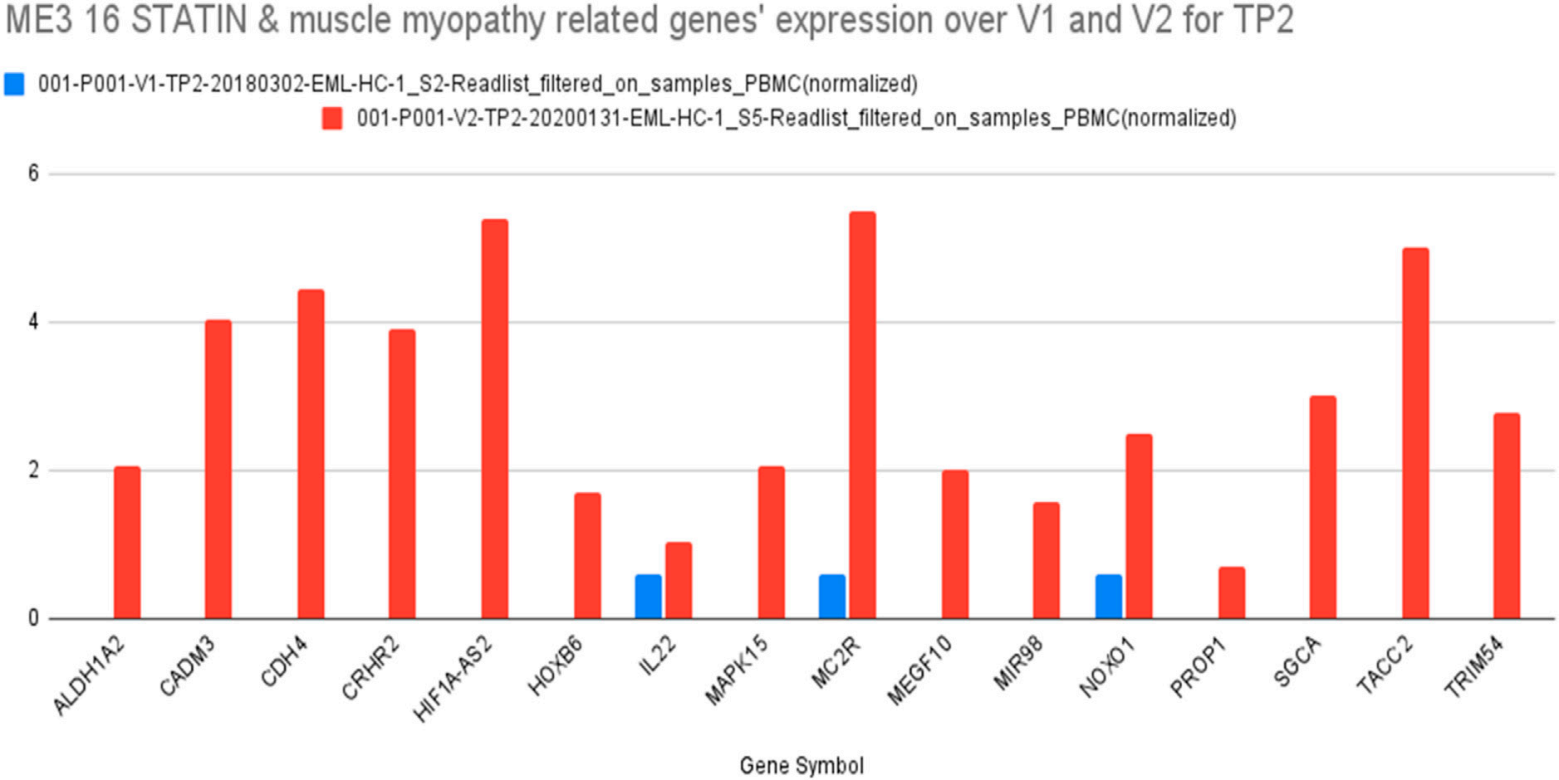
Sixteen genes in ME3 which are related to both STATIN and MYOPATHY and their differential expression at TP2 (V1, blue) and TP2 (V2, red) (details see text).

Based on further detailing of the gene ontology of these candidate genes and pathway analyses targeting known statin- and SAMS-associations, we identified 16 genes that were differentially expressed at peak exercise in full health (visit 1, TP2) compared with SAMS (visit 2, TP2) (Figure 6).

### Results 7

In order to investigate the biological relationship between these 16 Module 3 genes and those hypothetical models of SAMS-causation discussed in the Introduction, we conducted a systematic literature search of known properties of the 16 genes in relationship to the 8 hypotheses. The results are summarized in Table 2.

**TABLE 2:** Published literature on the 16 module 3 genes identified during our analyses and hypothetical SAMS-causation mechanisms.

Gene symbol	1. Depletion of cholesterol in muscle cell membranes	2. Reduction of intermediates of the cholesterol biosynthetic pathway	3. Reduction of seleno-protein synthesis	4. Inhibition of AKT/mTOR signaling pathways	5. Modification of ion-channel conductance	6. Genetic disposition	7. Modulation of mitochondrial function	8. Initiation of an auto-immune myopathy
ALDH1A2	No known muscle-membrane cholesterol link	Not linked	No	No	No	SNPs linked to hand osteoarthritis; developmental defects (Science) In neural tissues, ALDH1A2 regulates oxidative stress and may influence mitochondrial response (PMC) No autoimmune evidence		
CADM3	—	—	—	—	—	No clear data	—	—
CDH4	—	—	—	—	—	No clear data	—	—
CRHR2	—	—	—	CRHR2 signals via cAMP/PKA; AKT cross-talk possible but unproven	—	Polymorphisms studied in stress response traits	—	—
HIF1A-AS2	—	—	—	Modulates hypoxia-responsive HIF-1α signaling, which interfaces with mTOR regulation under low-oxygen stress	—	—	Hypoxia adaptation influences mitochondrial gene expression, Regulatory roles in hypoxia	—
HOXB6	—	—	—	Developmental TF; no direct AKT/mTOR connection	—	Rare congenital anomalies linked to HOXB6 dysregulation	—	—
IL22	—	—	Selenoproteins modulate IL-22 responses; IL-22 itself not part of synthesis machinery	IL-22 signals via STAT pathway with little known AKT/mTOR integration	—	Polymorphisms linked to inflammatory diseases	IL-22 implicated in inflammatory myopathies such as dermatomyositis	IL-22 is a cytokine involved in muscle-directed autoimmune inflammation
MAPK15	—	—	—	Atypical MAPK; plausible cross-talk with AKT/mTOR under stress contexts (theoretical)	—	—	—	—
MC2R	—	—	—	ACTH receptor activates cAMP/PKA; indirect AKT/mTOR cross-talk not established	—	Mutations cause familial glucocorticoid deficiency (endocrine)	—	—
MEGF10	—	—	—	—	—	Mutations cause congenital myopathy (EMARDD)	Mitochondrial dysfunction reported secondary to myopathy	Not autoimmune, but rare inflammation noted
MIR98	—	—	—	miR-98 reported to target PI3K/AKT components in cancer models (indirect)	—	—	—	—
NOXO1	—	—	—	NOXO1 organizes NADPH oxidase → ROS may perturb mTOR/AKT indirectly	—	No known genetic myopathy	ROS could affect mitochondrial function	No direct autoimmune studies
PROP1	—	—	—	Pituitary developmental TF(transcription factor); no reports of mTOR/AKT link	—	Mutations cause combined pituitary hormone deficiencies	—	—
SGCA	Sarcoglycan supports sarcolemma integrity; cholesterol depletion disrupts membranes broadly, but no direct link to SGCA	None described	None	Sarcoglycan complex components; dysfunction leads to muscular dystrophy (genetic predisposition)	Membrane disruption may influence ion channel localization	Mutations cause limb-girdle muscular dystrophy (LGMD2D/R3) with clear genetic predisposition	Secondary mitochondrial abnormalities in dystrophic muscle degeneration	While genetic, not autoimmune, inflammatory infiltration often occurs
TACC2	—	—	—	TACC2 involved in microtubule dynamics and cell cycle; limited link to mTOR	—	No clear genetic myopathy associations	—	—
TRIM54	—	—	—	Indirect via TRIM family mapping to AKT/mTOR pathways (not TRIM54 itself yet demonstrated) (Dove Medical Press, BioMed Central), TRIM54 causes myofibrillar myopathy in humans when mutated; — Mutations cause myofibrillar myopathy; autosomal inheritance; TRIM54 linked to mitochondrial DNA maintenance defects (PubMed, GeneCards) Mitochondrial features altered in patient cases No primary autoimmune role				

## Discussion

The main objective of this case report was to document a systematic analysis of differentially expressed PBMC genes, generated during standardized CPX-testing and analyze the resultant candidate genes with respect to their biological relationship to pathways hypothesized to be involved in causal SAMS mechanisms (Camerino et al., 2021).

Our main results are interesting: Based on pathway/reactome-analysis in StrandNGS, we identified–based on the known and published biology of the 16 genes described above, 5 pathway related to SAMS. These are: Beta-oxidation of very long chain fatty acids, Caspase activation via extrinsic apoptotic signalling pathway, TP53 Regulates Transcription of Genes Involved in Cytochrome C Release, RAC1 GTPase cycle (Indirect), RHO GTPases Activate NADPH Oxidases (Indirect) (Table 3).

**TABLE 3:** Effects of statins on relevant biochemical pathways.

Pathway List	Relevance
Beta-oxidation of very long chain fatty acids	Statins can impair mitochondrial function and fatty acid metabolism, contributing to mitochondrial dysfunction, which is a known mechanism in statin-induced myopathy. Beta-oxidation disruption can lead to energy deficits in muscle cells
Caspase activation via extrinsic apoptotic signalling pathway	Statins have been implicated in promoting apoptosis in muscle cells under some conditions, particularly when mitochondrial dysfunction or oxidative stress is present. This may contribute to muscle cell damage and myopathy
TP53 regulates transcription of genes involved in cytochrome C release	This is tied to the intrinsic apoptotic pathway, and statin-induced mitochondrial stress can lead to cytochrome c release and activation of apoptotic cascades. TP53 (p53) plays a key role in regulating this response
RAC1 GTPase cycle (Indirect)	RAC1 is involved in actin cytoskeleton dynamics, which may be affected by statins. There is also emerging evidence that RAC1 signaling can intersect with pathways influencing oxidative stress and cell survival in muscle tissue
RHO GTPases activate NADPH Oxidases (Indirect)	This can lead to ROS (reactive oxygen species) production, and oxidative stress is a known contributor to statin-associated muscle toxicity

The pathway of beta-oxidation of very long chain fatty acids is relevant within the SAMS-pathology concept because statins can impair mitochondrial function and fatty acid metabolism, contributing to mitochondrial dysfunction, which is a known mechanism in statin-induced myopathy. Beta-oxidation disruption can lead to energy deficits in muscle cells.

The pathway of caspase activation via extrinsic apoptotic signalling pathway is relevant within the SAMS-pathology concept because statins have been implicated in promoting apoptosis in muscle cells under some conditions, particularly when mitochondrial dysfunction or oxidative stress is present. This may contribute to muscle cell damage and myopathy.

The pathway of TP53 regulating transcription of genes involved in Cytochrome C release pathway is relevant within the SAMS-pathology concept because statins, via Cytochrome C release, are linked to the intrinsic apoptotic pathway, and statin-induced mitochondrial stress can lead to cytochrome c release and activation of apoptotic cascades. TP53 (p53) plays a key role in regulating this response.

The pathway of RAC1 GTPase cycle (Indirect) is relevant within the SAMS-pathology concept because statins, via RAC1 signalling, are involved in actin cytoskeleton dynamics. There is also emerging evidence that RAC1 signaling can intersect with pathways influencing oxidative stress and cell survival in muscle tissue.

The pathway of RHO GTPases Activating NADPH Oxidases (Indirect) is relevant within the SAMS-pathology concept because this can lead to reactive oxygen species (ROS) production, and oxidative stress is a known contributor to statin-associated muscle toxicity.

The involvement of these pathways is supported by the literature (Patel et al., 2015; Tiniakou et al., 2017; Llansó et al., 2024; Kim et al., 2019; Turner and Pirmohamed, 2019).

Since the auto-immune workup of the case did not add additional information, a discussion of the relationship of these candidate gene pathways to known auto-immune mechanisms will remain to be discussed in future studies using a comparable study design.

## Limitations

First, this case study is, as a single case report, limited by sample size. Yet in our opinion it provides a meaningful blueprint for the systematic pursuit of the analysis of SAMS-associated immune dysfunction mechanisms in future studies. Second, because of the rare constellation that the case study is published by the case study subject confers the risk of biased interpretation of findings. However, the team and co-authorship nature of the case study and the independent peer review process allow to maintain transparence and balance of the interpretation of the clinical and molecular data.

## Conclusion

This case study demonstrates that exercise-induced immune transcriptome profiling can reveal dynamic immunological changes associated with statin-induced myopathy. Our findings support the hypothesis of an immune-mediated component in SAMS and lay the groundwork for future studies to validate transcriptomic biomarkers for early detection and management.

## Data Availability

The data presented in the study are deposited in the ArrayExpress repository, accession number E-MTAB-15840.
